# Supplementary material for: Genetic manipulation of cell line derived reticulocytes enables dissection of host malaria invasion requirements
Source: Nat Commun. 2019 Aug 23;10:3806. doi: 10.1038/s41467-019-11790-w (PMC6707200; doi:10.1038/s41467-019-11790-w)
Supplement: Supplementary file 1 — Supplementary Information [file 41467_2019_11790_MOESM1_ESM.pdf]

Supplementary Information

Genetic manipulation of cell line derived reticulocytes enables dissection of host malaria invasion requirements

Satchwell et al

Supplementary Figure 1

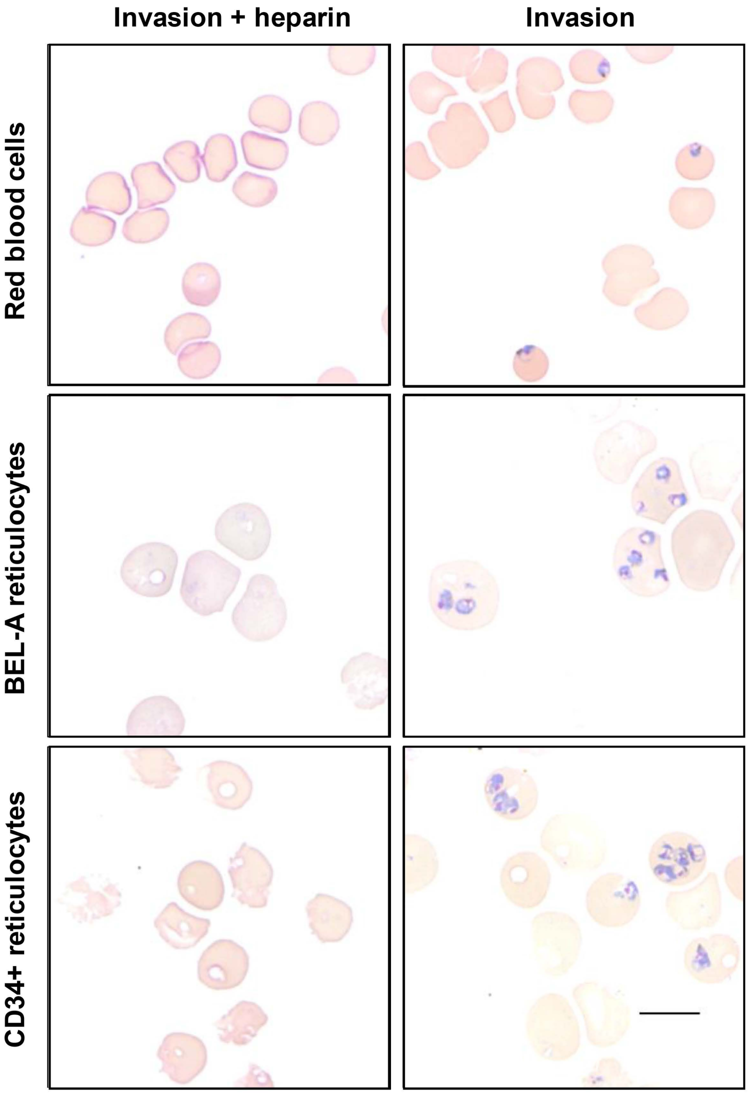

Reticulocytes derived from BEL-A or CD34⁺ cells exhibit higher parasite selectivity index than red blood cells. Giemsa stained cytospins of red blood cells, BEL-A and CD34⁺ cell derived reticulocytes illustrating instances of multiply invaded reticulocytes. Black scale bar shown at bottom right is 10 µm.

Supplementary Figure 2

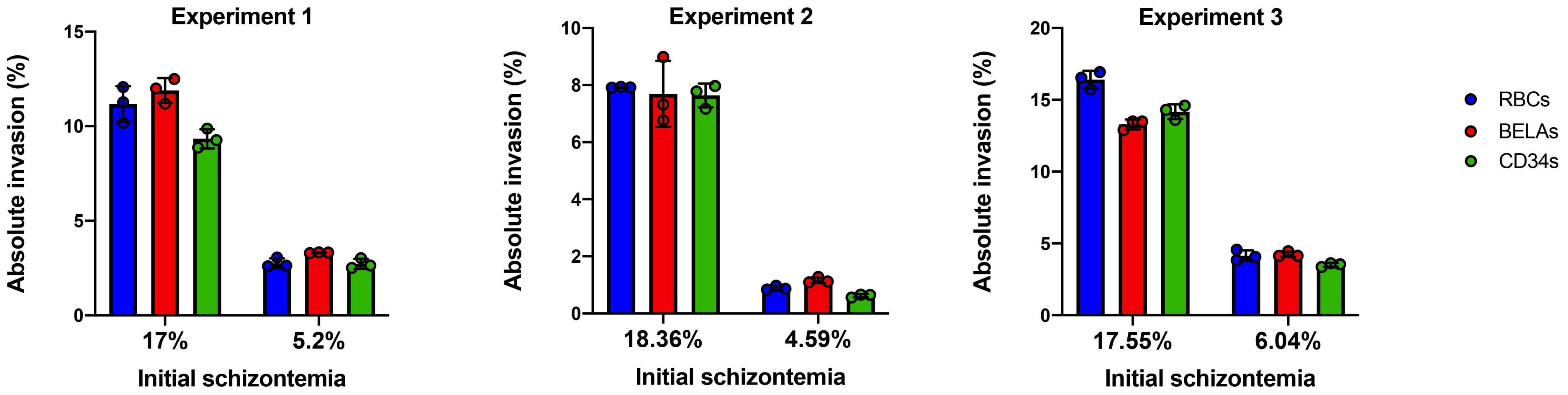

Efficiency of invasion into red blood cells, BEL-A and CD34+ cells at high and low multiplicities of infection (MOI). Invasion assays were set up at a high MOI (approximately 17% schizonts added) and a low MOI (approximately 5% schizonts added) in three independent experiments (n=3). The resultant ring stage parasitemia for red blood cells and $CD34^+$ and BEL-A derived reticulocytes, as measured by flow cytometry, is shown. The data are the mean and standard deviation of three biological replicates.

Supplementary Figure 3

A **Manual counting**

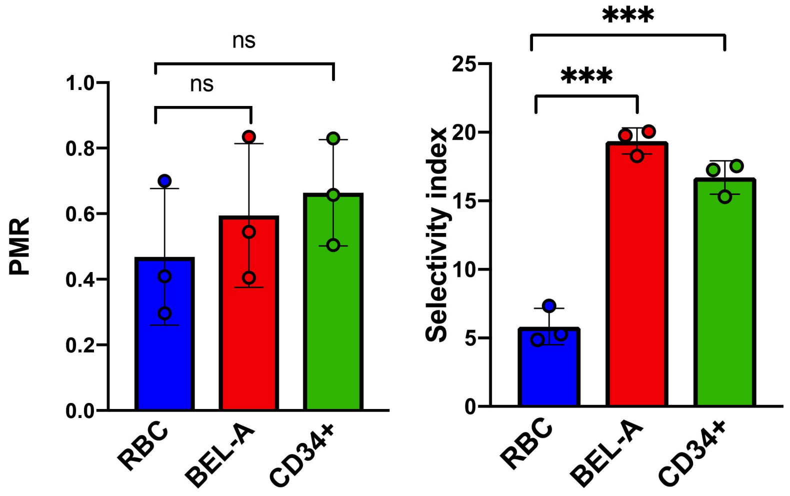

B **Flow cytometry**

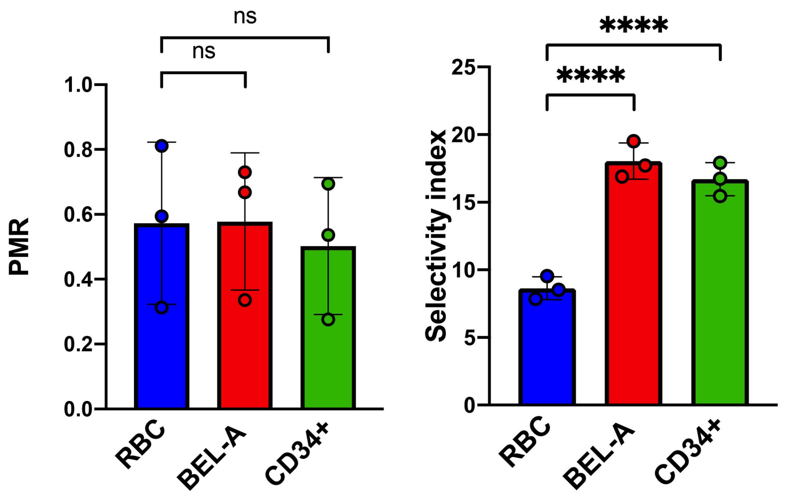

C

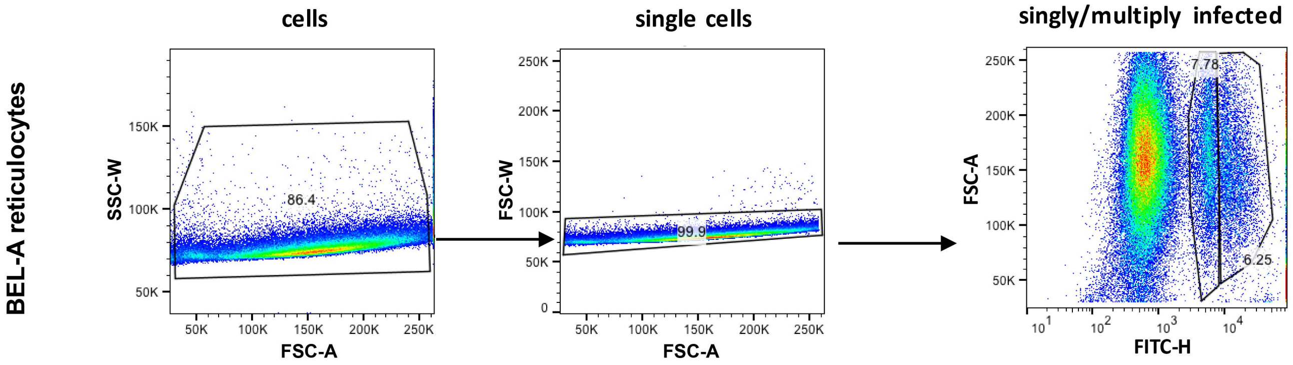

Comparison of parasite multiplication rate and selectivity index calculated by manual counting and flow cytometry. A) The parasite multiplication rate (PMR; left) and selectivity index (right) calculated by manual evaluation of Giemsa stained cytospins as presented in Figure 1C-D are reprinted here for comparison with the same parameters calculated by flow cytometry **(B).** The data are the mean and standard deviation of three biological replicates (n=3). A two-tailed t-test was used to calculate p values. Symbol ns indicates $p > 0.05$, *** indicates $p \leq 0.001$, and **** indicates $p \leq 0.0001$. Source data are provided as a Source Data file. **C)** Gating strategy used for flow cytometry analysis of all cell types, using data derived from BEL-A derived reticulocytes as an example.

Supplementary Figure 4

A

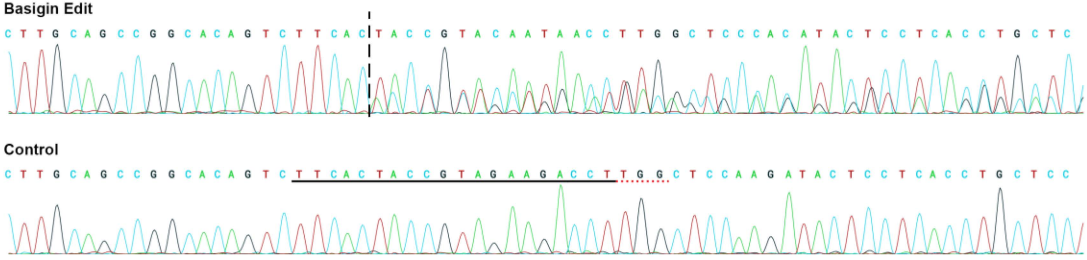

B

	Score	ICE Proposed Sequences
	0.6314	GCACAGTCTTCACTACCGTAGAAGA|nCCTTGGCTCCAAGATACTCCTCACCTGCTCCTTGAATGACAGCG
	0.0298	GCACAGTCTTCACT-----------|CCTTGGCTCCAAGATACTCCTCACCTGCTCCTTGAATGACAGCGC
	0.0271	GCACAGTCTTCAC------------|-----------AGATACTCCTCACCTGCTCCTTGAATGACAGCGC
	0.0231	GCACAGTCTTC--------------|--TTGGCTCCAAGATACTCCTCACCTGCTCCTTGAATGACAGCGC
	0.0216	GCACAGTCTTCAC------------|-CTTGGCTCCAAGATACTCCTCACCTGCTCCTTGAATGACAGCGC
	0.0166	GCACAGTCTTCACTACCG-------|--------CCAAGATACTCCTCACCTGCTCCTTGAATGACAGCGC
	0.0119	GCACAGTCTTCACTACCG-------|-CTTGGCTCCAAGATACTCCTCACCTGCTCCTTGAATGACAGCGC
	0.0119	GCACAGTCTTCACTACCG-------|CCTTGGCTCCAAGATACTCCTCACCTGCTCCTTGAATGACAGCGC
	0.0108	GCACAGTCTTCAC------------|---------------ACTCCTCACCTGCTCCTTGAATGACAGCGC
	0.0071	GCACAGTCTTCACTACCGTAGAAGA|nnnnnCCTTGGCTCCAAGATACTCCTCACCTGCTCCTTGAATGAC
	0.0069	GCACAGTCTTCAC------------|----GGCTCCAAGATACTCCTCACCTGCTCCTTGAATGACAGCGC
	0.0069	GCACAGTCTTC--------------|---------------ACTCCTCACCTGCTCCTTGAATGACAGCGC
	0.0063	GCACAGTCTTCACTAC---------|---------------ACTCCTCACCTGCTCCTTGAATGACAGCGC
	0.0062	GCACAGTCTTCAC------------|CCTTGGCTCCAAGATACTCCTCACCTGCTCCTTGAATGACAGCGC
	0.0056	GCACAGTCTT---------------|---------------ACTCCTCACCTGCTCCTTGAATGACAGCGC
	0.0047	GCACAGTCTTCACTACCGT------|----GGCTCCAAGATACTCCTCACCTGCTCCTTGAATGACAGCGC
	0.0046	GCACAGTCTTCAC------------|--------------TACTCCTCACCTGCTCCTTGAATGACAGCGC
	0.0040	GCACAGTCTTCAC------------|-----GCTCCAAGATACTCCTCACCTGCTCCTTGAATGACAGCGC
	0.0010	GCACAGTCTT---------------|--------------TACTCCTCACCTGCTCCTTGAATGACAGCGC
WT	0.0000	GCACAGTCTTCACTACCGTAGAAGA|CCTTGGCTCCAAGATACTCCTCACCTGCTCCTTGAATGACAGCGC

CRISPR-edited BSG KO BEL-A cell line contains compound heterozygous BSG mutations. A) Sanger sequencing of clonal edited line shows mixed spectra emerging (vertical dashed line) within the vicinity of the gRNA target site (horizontal line), indicating a heterozygous edit. **B)** ICE analysis was unable to infer any wild-type sequence, indicating a compound heterozygous edit. Regression scores indicate likelihood of computationally generated proposed sequences in sample. Wild-type sequence is shown in red with a regression score of 0.

Supplementary Figure 5

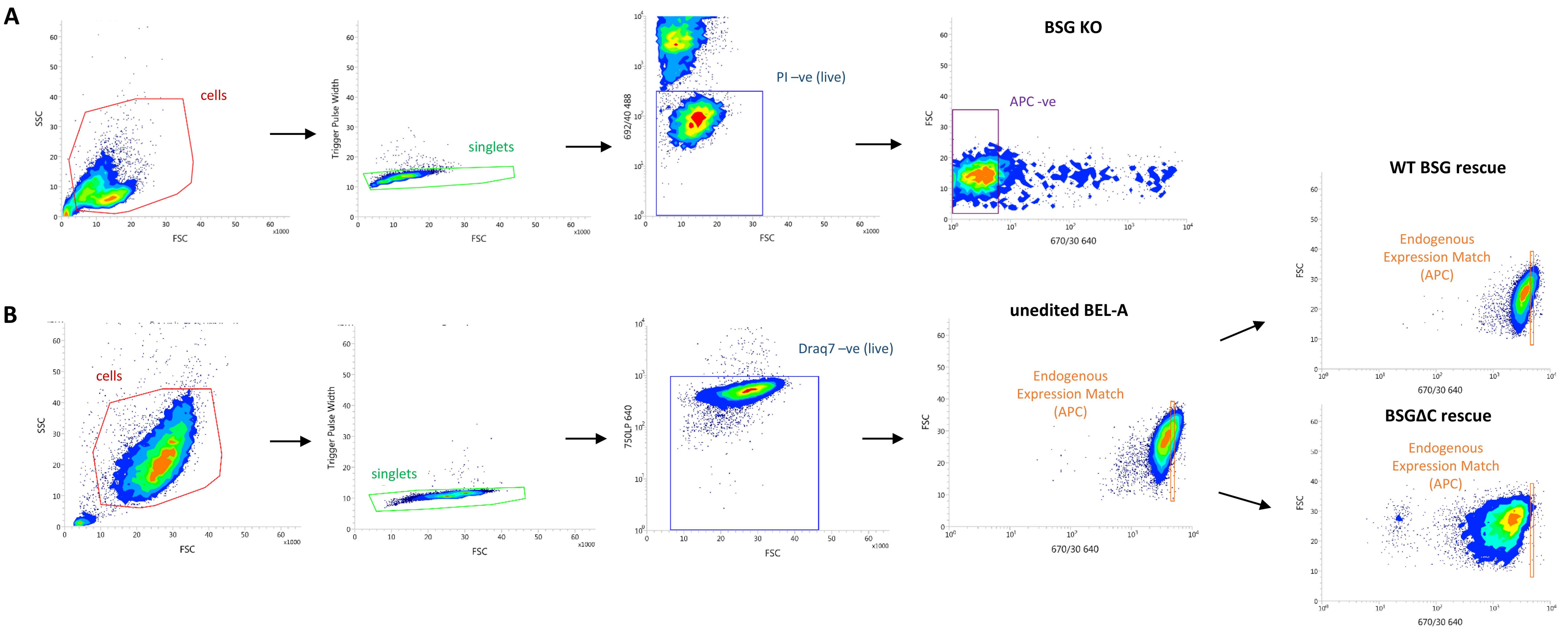

Sorting strategies for derivation of BSG KO and rescue clones. A) Expanding BEL-A cells labelled with anti-basigin antibody HIM6 and APC conjugated secondary antibody also stained with PI were isolated according to a sorting strategy that derives viable (PI negative), basigin (HIM6) negative clones **B)** Single clones of viable (Draq7 negative) BSG KO cells rescued with WT BSG or BSGΔC were sorted according to the indicated gate for matching to endogenous expression of basigin (HIM6) on unedited BEL-A cells.

Supplementary Figure 6

A

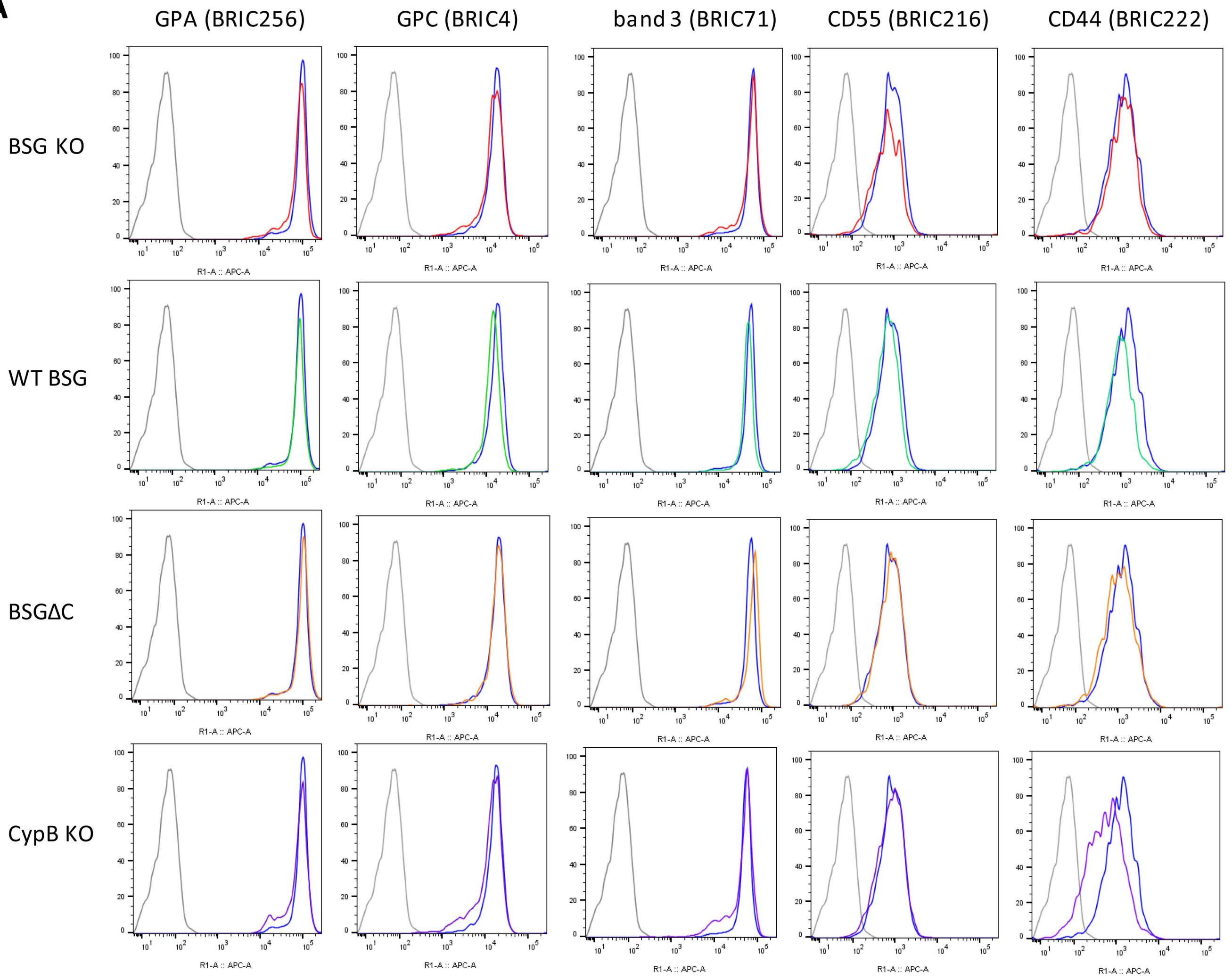

B

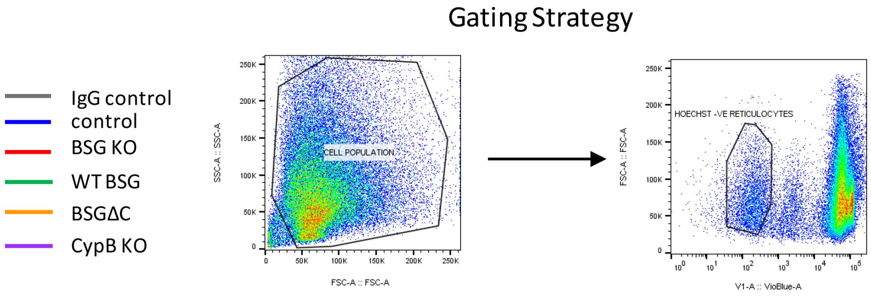

BSG KO does not reduce reticulocyte expression of other parasite associated erythrocyte surface receptors. **A)** Flow cytometry histograms illustrating expression of indicated malaria receptors in reticulocytes derived from BSG KO, WT BSG, BSGΔC and CypB KO BEL-A cell lines compared to unedited BEL-A derived reticulocytes. **B)** Gating strategy for identification of Hoechst negative reticulocytes within a mixed erythroid culture population

Supplementary Figure 7

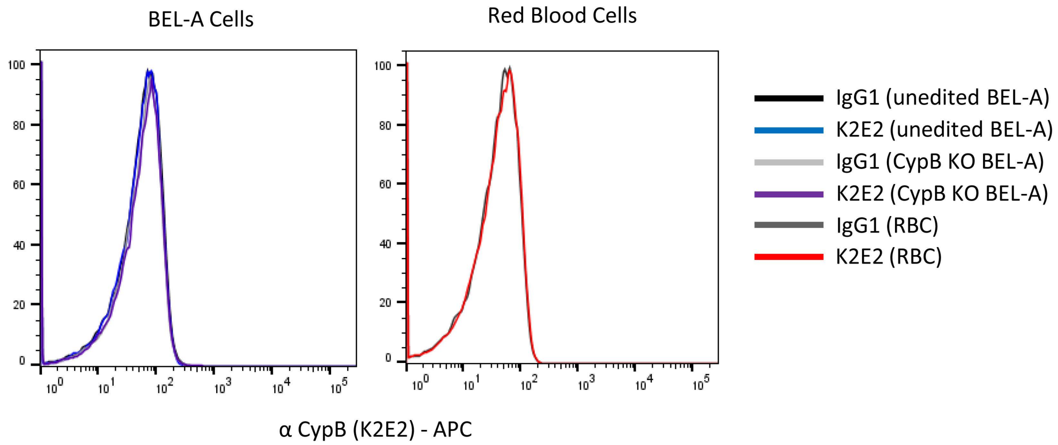

Cyclophilin B is undetectable at the cell surface of red blood cells or BEL-A cells. Flow cytometry histograms illustrating absence of detectable cyclophilin B in BEL-A cells and red blood cells using the monoclonal antibody K2E2.

Supplementary Figure 8

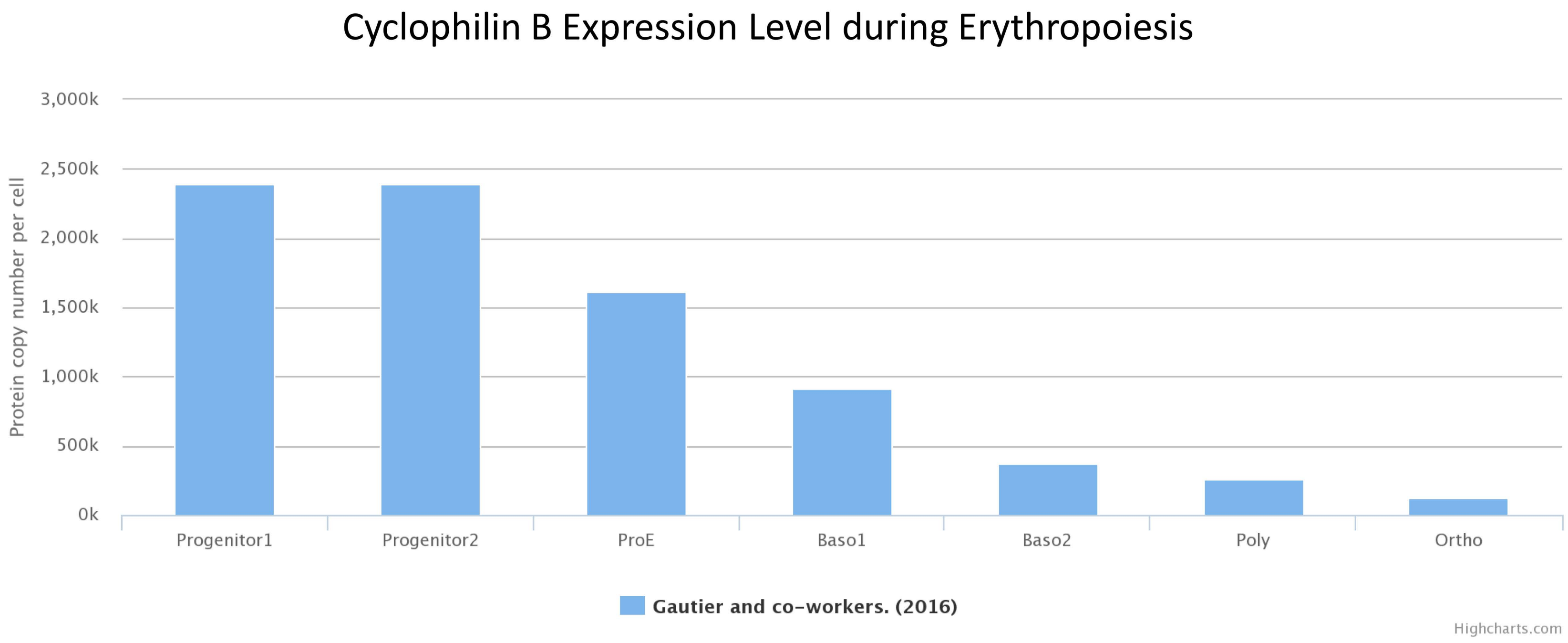

Cyclophilin B protein expression is reduced during erythropoiesis. Bar chart illustrating a progressive and dramatic reduction in cyclophilin B copy number per cell during terminal erythroid differentiation of primary hematopoietic stem cells. Figure was generated using the proteomic dataset and database published by Gautier and colleagues (Gautier et al, Cell Reports 2016)

Supplementary Figure 9

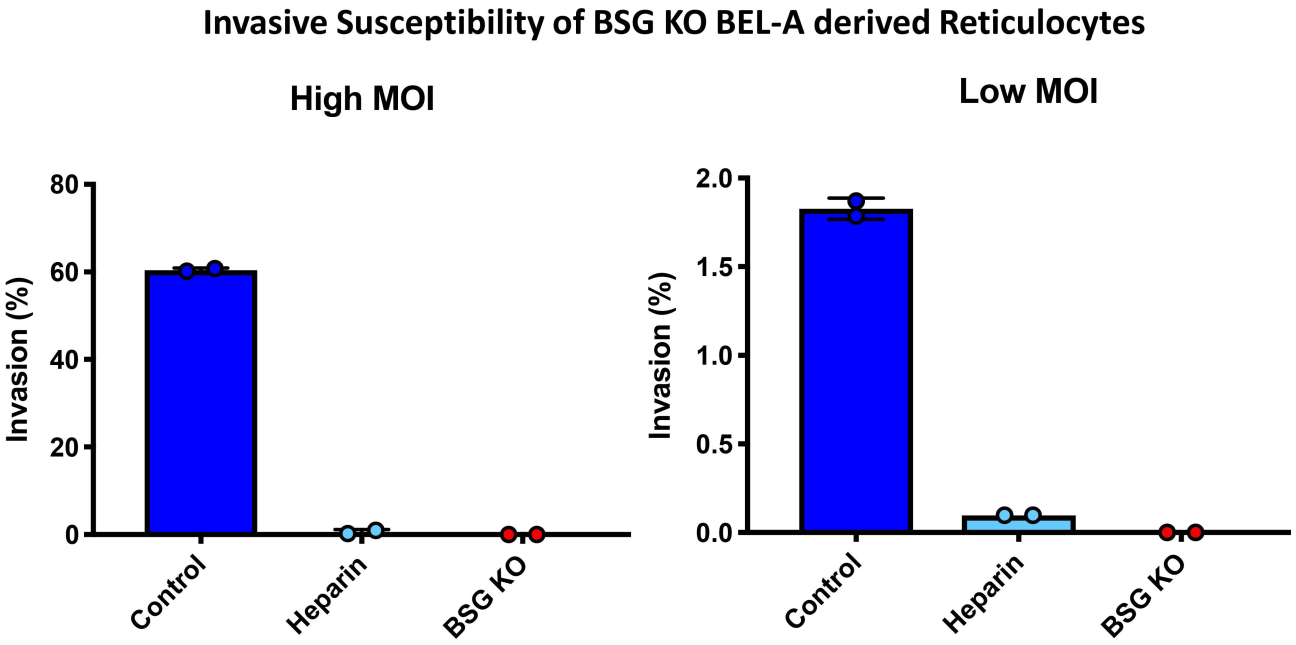

BSG KO reticulocytes are refractory to invasion even at high multiplicity of infection. Histogram illustrating percentage invasion of reticulocytes derived from unedited and BSG KO reticulocytes at high and low MOI. Invasion efficiencies are calculated based on two independent manual counts of rings in Giemsa stained cytospins. Error bars represent standard deviation of the mean. Source data are provided as a Source Data file.

Supplementary Figure 10

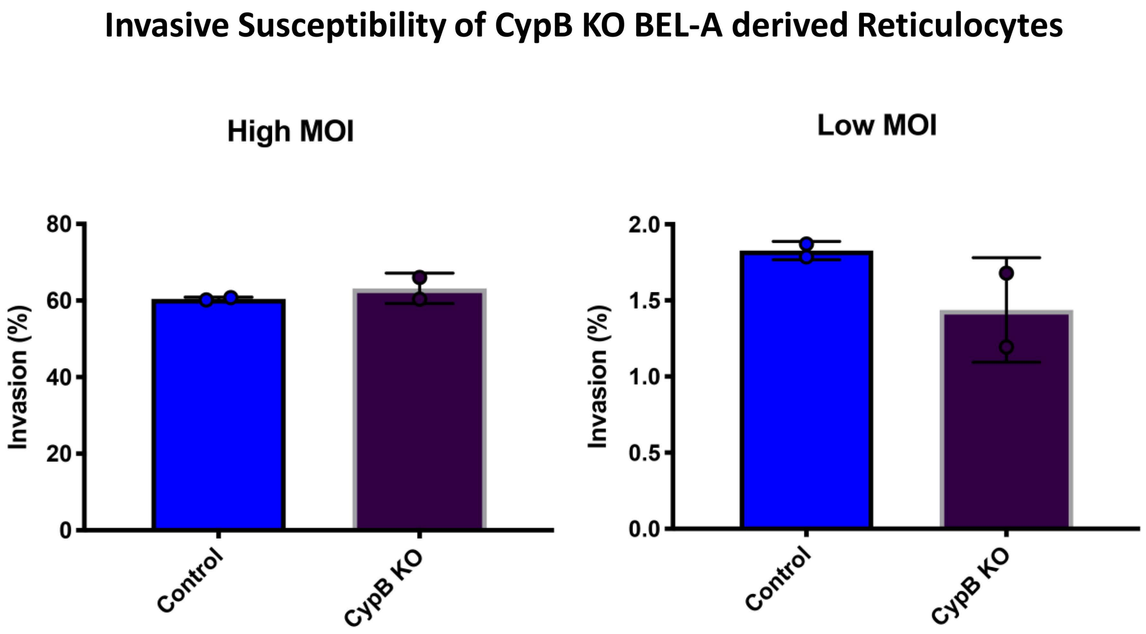

CypB KO does not affect susceptibility to invasion by *P.falciparum* Histogram illustrating percentage invasion of reticulocytes derived from unedited and CypB KO reticulocytes at high and low MOI. Invasion efficiencies are calculated based on two independent manual counts of rings in Giemsa stained cytospins. Error bars represent standard deviation of the mean. Source data are provided as a Source Data file.
