# Supplementary material for: Genetic manipulation of cell line derived reticulocytes enables dissection of host malaria invasion requirements
Source: Nat Commun. 2019 Aug 23;10:3806. doi: 10.1038/s41467-019-11790-w (PMC6707200; doi:10.1038/s41467-019-11790-w)
Supplement: Supplementary file 3 — Reporting Summary [file 41467_2019_11790_MOESM3_ESM.pdf]

nature research

Corresponding author(s): Timothy J Satchwell
Katherine E Wright
Jake Baum

Last updated by author(s): Jul 26, 2019

Reporting Summary

Nature Research wishes to improve the reproducibility of the work that we publish. This form provides structure for consistency and transparency in reporting. For further information on Nature Research policies, see Authors & Referees and the Editorial Policy Checklist .

Statistics

For all statistical analyses, confirm that the following items are present in the figure legend, table legend, main text, or Methods section.

n/a	Confirmed	
☐	☒	The exact sample size (*n*) for each experimental group/condition, given as a discrete number and unit of measurement
☐	☒	A statement on whether measurements were taken from distinct samples or whether the same sample was measured repeatedly
☐	☒	The statistical test(s) used AND whether they are one- or two-sided<br>*Only common tests should be described solely by name; describe more complex techniques in the Methods section.*
☒	☐	A description of all covariates tested
☒	☐	A description of any assumptions or corrections, such as tests of normality and adjustment for multiple comparisons
☐	☒	A full description of the statistical parameters including central tendency (e.g. means) or other basic estimates (e.g. regression coefficient) AND variation (e.g. standard deviation) or associated estimates of uncertainty (e.g. confidence intervals)
☐	☒	For null hypothesis testing, the test statistic (e.g. *F*, *t*, *r*) with confidence intervals, effect sizes, degrees of freedom and *P* value noted<br>*Give P values as exact values whenever suitable.*
☒	☐	For Bayesian analysis, information on the choice of priors and Markov chain Monte Carlo settings
☒	☐	For hierarchical and complex designs, identification of the appropriate level for tests and full reporting of outcomes
☒	☐	Estimates of effect sizes (e.g. Cohen's *d*, Pearson's *r*), indicating how they were calculated

Our web collection on statistics for biologists contains articles on many of the points above.

Software and code

Policy information about availability of computer code

Data collection	No software was used for data collection
Data analysis	Flow cytometry data were analysed using FlowJo<br>Inference of CRISPR Edits (ICE) was used for analysis of Sanger sequence data (reference Hsiau T, M. T., Waite K, JYang J, Kelso R, Holden K, Stoner R. Inference of CRISPR Edits from Sanger Trace Data. bioRxiv, doi: https://doi.org/10.1101/251082 (2018).)<br>Statistics were calculated with GraphPad Prism 7.

For manuscripts utilizing custom algorithms or software that are central to the research but not yet described in published literature, software must be made available to editors/reviewers. We strongly encourage code deposition in a community repository (e.g. GitHub). See the Nature Research guidelines for submitting code & software for further information.

Data

Policy information about availability of data

All manuscripts must include a data availability statement. This statement should provide the following information, where applicable:

- Accession codes, unique identifiers, or web links for publicly available datasets
- A list of figures that have associated raw data
- A description of any restrictions on data availability

The data that support the findings of this study are available from the corresponding author upon reasonable request.

Field-specific reporting

Please select the one below that is the best fit for your research. If you are not sure, read the appropriate sections before making your selection.

Life sciences study design

All studies must disclose on these points even when the disclosure is negative.

Sample size	The sample size for invasion assays was determined by the yield of BEL-A derived reticulocytes.
Data exclusions	No data were excluded from the analysis.
Replication	The invasion assays were repeated a total of three times successfully.
Randomization	Randomization was not relevant to the study.
Blinding	Cells were counted on slides by two researchers independently and blindly.

Reporting for specific materials, systems and methods

We require information from authors about some types of materials, experimental systems and methods used in many studies. Here, indicate whether each material, system or method listed is relevant to your study. If you are not sure if a list item applies to your research, read the appropriate section before selecting a response.

Materials & experimental systems

n/a	Involved in the study	
☐	☒	Antibodies
☐	☒	Eukaryotic cell lines
☒	☐	Palaeontology
☒	☐	Animals and other organisms
☒	☐	Human research participants
☒	☐	Clinical data

Methods

n/a	Involved in the study	
☒	☐	ChIP-seq
☐	☒	Flow cytometry
☒	☐	MRI-based neuroimaging

Antibodies

Antibodies used	Mouse monoclonal antibodies used were as follows: BRIC4 (GPC), BRIC216 (CD55), BRIC222 (CD44), BRIC71 (band 3), BRIC256 (GPA) (all IBGRL hybridoma supernatants used 1:2), HIM6 (basigin) (Biolegend 1:50 flow cytometry, 1:500 immunoblotting), ab64616 (basigin C-terminal) (AbCam, 1:500)), K2E2 (CypB) (Santa Cruz, (1:50 flow cytometry, 1:500 immunoblotting), SAB2101856 (CypB N-terminal) (Sigma 1:500), GAPDH 0411 (Santa Cruz) (1:1000), IgG1 control MG1-45 (1:50 Biolegend). Secondary antibodies were APC conjugated monoclonal rat anti-mouse IgG1 RMG1-1 (Biolegend 1:50), swine anti-rabbit HRP (P0399) or rabbit anti-mouse HRP (P0260) (Dako 1:2000).
Validation	Validations are available on IBGRL, Biolegend and Santa Cruz websites for indicated antibody clones

Eukaryotic cell lines

Policy information about cell lines

Cell line source(s)	Bristol Erythroid Line Adult (Jan Frayne)
Authentication	BEL-A cell line was recently published Trakarnsanga et al, 2017 Nat Commun 8: 14750
Mycoplasma contamination	Cell line was not tested for mycoplasma contamination
Commonly misidentified lines (See ICLAC register)	*Name any commonly misidentified cell lines used in the study and provide a rationale for their use.*

Flow Cytometry

Plots

Confirm that:

- [x] The axis labels state the marker and fluorochrome used (e.g. CD4-FITC).
- [x] The axis scales are clearly visible. Include numbers along axes only for bottom left plot of group (a 'group' is an analysis of identical markers).
- [x] All plots are contour plots with outliers or pseudocolor plots.
- [x] A numerical value for number of cells or percentage (with statistics) is provided.

Methodology

Sample preparation	Sample preparation is described in manuscript methods.<br>For flow cytometry on undifferentiated BEL-As, 1 × 105 cells resuspended in PBSAG (PBS + 1 mg/ml BSA, 2 mg/ml glucose) + 1% BSA were labelled with primary antibody for 30 min at 4°C. Cells were washed in PBSAG, incubated for 30 min at 4°C with appropriate APC-conjugated secondary antibody, and washed and data acquired on a MacsQuant VYB Analyser using a plate reader. For differentiated BEL-As, cells were stained with 5 µg/ml Hoechst 33342 then fixed if required in 1% paraformaldehyde, 0.0075% glutaraldehyde to reduce antibody binding-induced agglutination before labelling with antibodies as described. Reticulocytes were identified by gating upon Hoechst-negative population.<br>For flow cytometry, cells were washed in PBSAG, stained with SybrGreen (1:1000 in PBS; Sigma-Aldrich) for 20 minutes at room temperature in the dark, and washed three times in PBSAG. 1 x105 cells from each well were acquired using the FITC channel of a BD Fortessa flow cytometer.
Instrument	MACSQuant or BD Fortessa
Software	Data were analysed using FlowJo v10
Cell population abundance	*Describe the abundance of the relevant cell populations within post-sort fractions, providing details on the purity of the samples and how it was determined.*
Gating strategy	Gating on reticulocytes within mixed population of end stage cultures for characterisation of red blood cell protein expression was based on Hoechst33342 negativity. Gating strategy for identification of invaded reticulocytes is shown in the manuscript and is based upon positivity for SYBR green

- [x] Tick this box to confirm that a figure exemplifying the gating strategy is provided in the Supplementary Information.
